# Efficient Recombinant Production in Mammalian Cells Using a Novel IR/MAR Gene Amplification Method

**DOI:** 10.1371/journal.pone.0041787

**Published:** 2012-07-23

**Authors:** Yoshio Araki, Tetsuro Hamafuji, Chiemi Noguchi, Noriaki Shimizu

**Affiliations:** 1 Graduate School of Biosphere Science, Hiroshima University, Higashi-hiroshima, Hiroshima, Japan; 2 Transgenic Inc. 7-1-14, Minatojimaminami-machi, Chuo-ku, Kobe, Japan; Naval Research Laboratory, United States of America

## Abstract

We previously found that plasmids bearing a mammalian replication initiation region (IR) and a nuclear matrix attachment region (MAR) efficiently initiate gene amplification and spontaneously increase their copy numbers in animal cells. In this study, this novel method was applied to the establishment of cells with high recombinant antibody production. The level of recombinant antibody expression was tightly correlated with the efficiency of plasmid amplification and the cytogenetic appearance of the amplified genes, and was strongly dependent on cell type. By using a widely used cell line for industrial protein production, CHO DG44, clones expressing very high levels of antibody were easily obtained. High-producer clones stably expressed the antibody over several months without eliciting changes in both the protein expression level and the cytogenetic appearance of the amplified genes. The integrity and reactivity of the protein produced by this method was fine. In serum-free suspension culture, the specific protein production rate in high-density cultures was 29.4 pg/cell/day. In conclusion, the IR/MAR gene amplification method is a novel and efficient platform for recombinant antibody production in mammalian cells, which rapidly and easily enables the establishment of stable high-producer cell clone.

## Introduction

Production of recombinant proteins in cultured mammalian cells is becoming more critical as the need for large amounts of pharmaceuticals protein, e.g. humanized antibody, is increasing rapidly. Large-scale culture of mammalian cells is more expensive and technically challenging than that of yeast or bacterial cells. However, patterns of protein folding and protein modification, such as glycosylation, are specific to mammalian cells, and bacterial and yeast proteins may invoke immune responses in humans. Furthermore, the presence of trace amounts of yeast or bacterial components in preparations of proteins for therapeutic use is unacceptable. Therefore, proteins for therapeutic use must be produced in mammalian cells. For industrial protein production, the most popular mammalian cell has been the Chinese hamster ovary (CHO) cell line and its derivatives. Industrial production of recombinant protein in these cells is a multi-part process and entails the development of high-producer cells, culture of the cells at high density in chemically defined medium, and purification of the target protein (reviewed in [1]). Here, we describe an improvement in the first step of this process with the introduction of a novel gene amplification method that efficiently increases target gene copy number in the cultured cells.

Amplification of oncogenes or drug-resistance genes has frequently been associated with the malignant transformation of human cells, where gene amplification induces overproduction of the cognate protein product. Therefore, the induction of target gene amplification has often been used to generate cells that produce high levels of a target for the pharmaceutical industry. A frequently used method for target gene amplification is the linkage of the dihyfrofolate reductase (DHFR) gene to the target gene, followed by amplification induced by increasing concentrations of the DHFR inhibitor methotrexate (MTx) in a DHFR-deficient CHO subline, such as DG44. However, this method is time- and labor-intensive [2], usually requiring more than six months for a skilled technician to complete. Furthermore, the high-producer cells produced by this method are frequently unstable [3], and the structural integrity and productivity of the transgene often declines rapidly. Such instability was also reported for another gene amplification-mediated method (GS/MSX method; [4], [5]). Therefore, an alternative method that enables rapid and efficient acquisition of stable high-producer cell is strongly required [1].

As an alternative to this approach, we previously developed a new method that amplifies any gene in mammalian cells [6], [7]. The method utilizes a plasmid that has a mammalian replication initiation region (IR) and a nuclear matrix attachment region (MAR); thus, we refer to the technique as IR/MAR gene amplification. When this plasmid was introduced into human colorectal carcinoma COLO 320 cells, a pool of stable transfectants was obtained after selecting for plasmid-coded drug-resistance to a drug such as blasticidin. Fluorescence in situ hybridization (FISH) resulted in a bright signal for the highly amplified sequence in the transfectants, and these signals located at either extrachromosomal double minutes (DMs) or chromosomal homogeneously staining regions (HSR), whose appearance was very close to the one that was generated during human malignant transformation. The method is simple, rapid, and highly effective, generating DMs or HSRs bearing thousands of copies of transgenes per human COLO 320 cell in more than 80% of the transfectants within about one month. Presence of both IR and MAR sequences in the plasmid was required for the efficient amplification [6], [7], and deletion of either of which resulted in the great reduction of the gene amplification efficiency. It may be related to that the replication initiation in mammalian cells requires attachment to the nuclear matrix [8], [9]. Furthermore, unrelated sequence with similar in length to IR could not support the gene amplification [10]. On the other hand, there were reports that MAR [11]–[14], IR [15], anti-repressor elements [16] or chromatin opening elements [17] enhanced expression from the flanking target gene, and it was applied to the recombinant protein production. It was suggested that these sequences reduced the effect of heterochromatin that might flank the chromosomal integration site. However, these methods did not result in gene amplification, presumably because spontaneous gene amplification requires both IR and MAR, as described in above.

We have uncovered the mechanism of gene amplification mediated by the IR/MAR plasmid [7], [18], [19]. This method has also been used to investigate the behavior of extrachromosomal DMs during cell cycle progression [20]–[24], and several nuclear function [10], [20], [25]–[27]. In addition, this method is effective for the overproduction of green fluorescent protein (GFP) [20] and human cyclooxygenase-1 (hCOX-1) [28]. We now report the efficient production of a human antibody by this method. We show the method is successful and superior to the conventional DHFR/MTx method in many respects, i.e. simplicity, rapidity, productivity, and stability of the established clones.

## Materials and Methods

### Plasmid Construction

**Figure 1:**
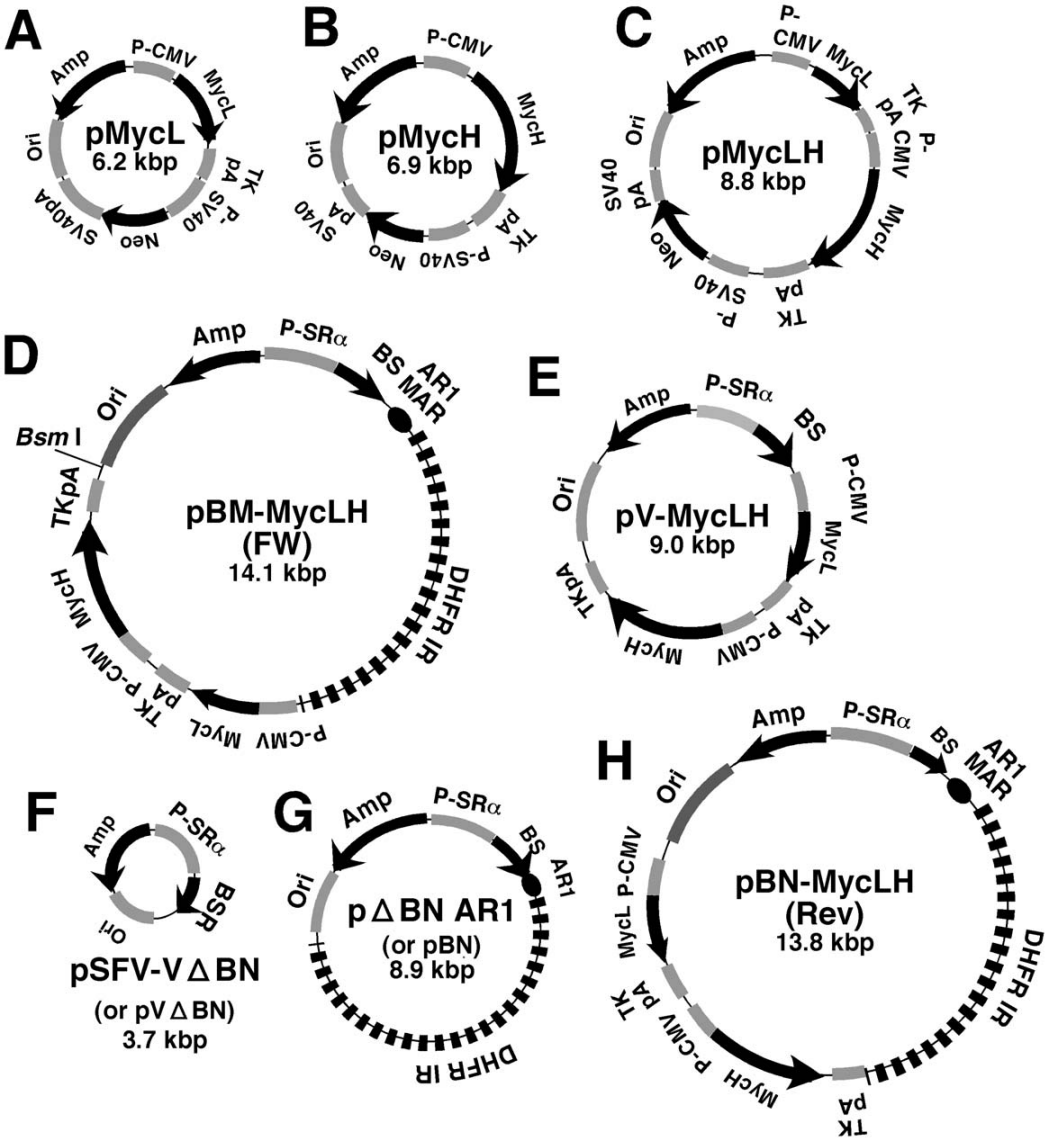
Plasmids used in this study. The schematic structures are illustrated in this Figure. The black arrow represents gene and its orientation. An IR from *DHFR* non-coding region and an AR1 MAR from *Ig κ* intron are illustrated as dashed bold line and an oval respectively in D, G and H.

The structures of the plasmids used in this study are shown in Figure 1. The plasmid expressing anti-c-MYC (9E10) humanized antibody was constructed as described below. The mouse hybridoma MYC 1-9E10.2 cell line was obtained from ATCC (American Type Culture Collection; CRL-1729). RNA was isolated from these cells and converted to cDNA by reverse transcription. The heavy (H) and light (L) chain variable region sequences were amplified by PCR and the products were ligated to the H-chain and L-chain constant region sequences, respectively, of human immunoglobulin (Ig) G1, which was obtained from MGC (Mammalian Gene Collection). The sequences of the 9E10 antibody and human IgG1 genes were obtained from NCBI (National Center for Biotechnology Information). The *Ig κ* signal sequence was obtained from plasmid pSectag2A (Invitrogen), and placed at the 5′ end of the anti-c-Myc H- and L-chain genes. The open reading frame, consisting of the signal sequence and variable or constant region sequences of the H- and L-chains, was inserted into pcDNA3.3 TOPO (Invitrogen) under the control of the CMV promoter. The plasmids expressing the L- and H-chains of humanized anti-c-MYC (9E10) antibody (pMyc L and pMyc H) are shown in Fig. 1A and 1B. The plasmid expressing both L- and H-chains (pMycLH; Fig. 1C) was constructed by PCR amplification of the entire expression cassette for the L-chain gene (including the promoter, coding sequence, and TK polyA sequence), followed by insertion into the 5′ end of H-chain gene in pMycH in the same orientation, using “In-Fusion® HD Cloning Kit w/Cloning Enhancer” (Invitrogene; code #639633).

The plasmid pΔBN.AR1 (pBN, Fig. 1G) was described in a previous publication [7]. To construct pBM-MycLH (Fig. 1D), plasmid pΔB.AR1 [7] was digested with *Bam* HI and *Mlu* I to remove the hygromycin gene expression cassette, which was replaced by a synthetic oligonucleotide comprising a multiple cloning site (MCS). The resulting plasmid (pΔBM.AR1 MCS) was then digested with *Swa* I to excise the MCS, followed by ligation of the product to the *Nru* I/*Psi* I fragment of pMycLH, containing the H- and the L-chain gene expression cassettes. For pBN-MycLH, plasmid pΔBN.AR1was digested with *Bam* HI, blunted, and ligated to the *Nru* I/*Psi* I fragment of pMycLH. The resulting plasmid contained the H- and L-chain genes, either in the same (FW) or reverse (Rev) orientation with respect to the blasticidin resistance R (BS) gene. pBM-MycLH (FW) was frequently abbreviated as simply pBM-MycLH. For the construction of pV-MycLH (Fig. 1E), the larger fragment of the *Bss* HI/*Kpn* I digest of pBM-MycLH(FW), containing the L- and H-chain gene cassettes, *Ori* in *E. coli*, Amp, and a portion of the BS gene, was used. The DNA was mixed with the PCR product that contained the remaining portion of the BS gene and the 15- bp sequence homologous to the end of *Bss* HI/*Kpn* I digest of pBM-MycLH. This DNA was circularized using the “In-Fusion® HD Cloning Kit w/Cloning Enhancer”. For the construction of pSFV-V ΔBN (pV ΔBN) (Fig. 1F), the plasmid pSFV-V [6] was digested with *Bam* HI/*Nru* I, and the fragments obtained were blunted with KOD polymerase and circularized by self-ligation. Plasmid DNA encoding either the CAG promoter (RDB2546; pxCAG) or the EF1α promoter (RDB5215; pAxEFwtit2) was obtained from RIKEN DNA Bank. The promoter sequences were inserted in place of the CAG promoter in pMycL and pMycH by PCR amplification and the In-Fusion® reaction. All constructed plasmids were verified by sequencing the junctions between the inserted fragments.

**Figure 2:**
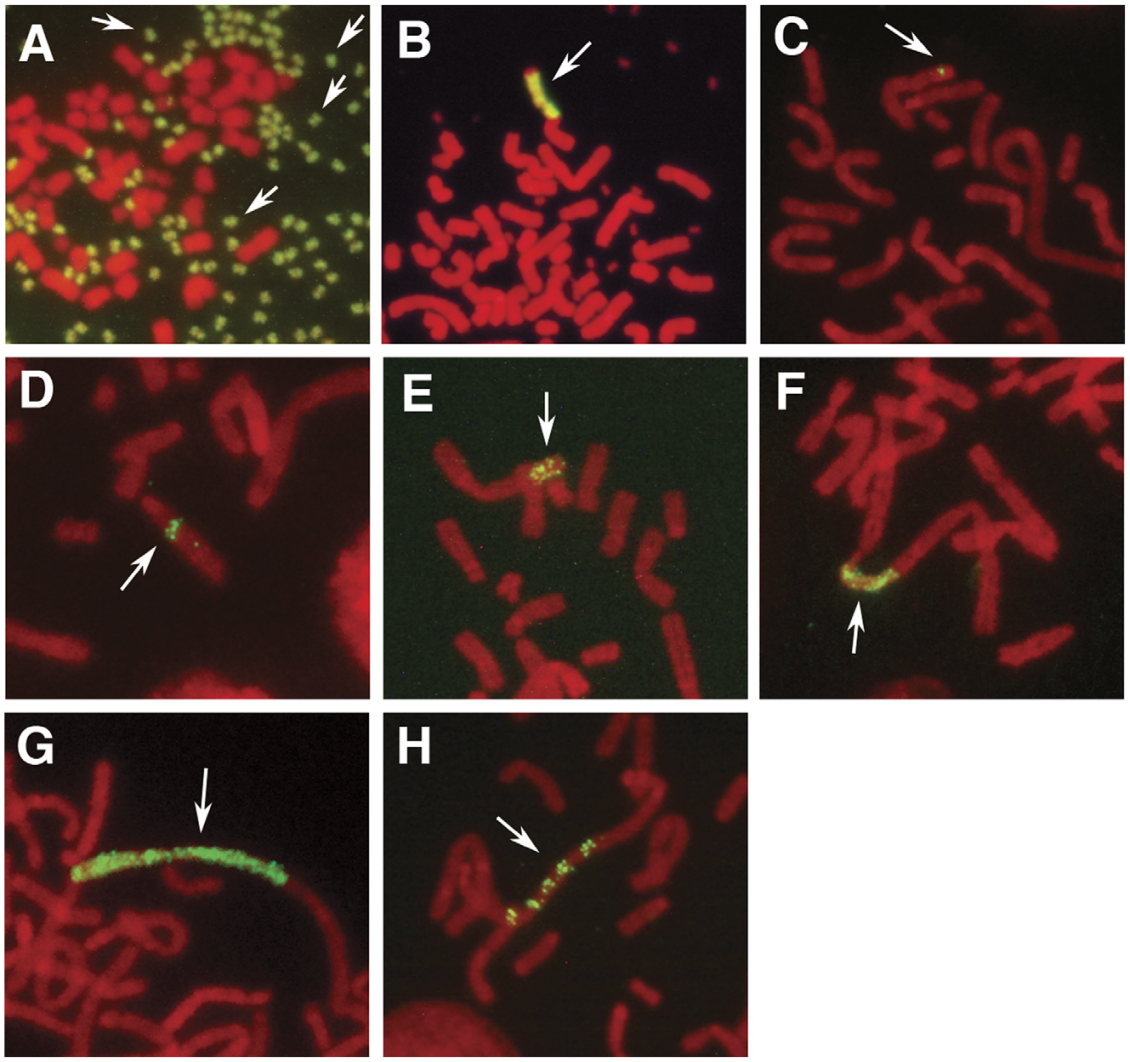
Generation of several kinds of amplified structures by the IR/MAR plasmid depending on the cell type. The IR/MAR plasmid was transfected into cells, a stable transformant was selected by drug selection, and a metaphase chromosome spread was prepared. Green fluorescence indicates the plasmid sequence after FISH and red fluorescence indicates the chromosome after propidium iodide (PI) staining. Representative images of several types of gene amplification are shown. In COLO 320DM cells, the plasmid was usually amplified as extrachromosomal DMs (A) or an chromosomal homogeneous array of HSR (B). In CHO DG44 cells (C to H), the IR/MAR plasmid was amplified as a chromosomal structure of varying length, including a tiny dot (C), a line (D) and a HSR longer than the metaphase chromosome width (cw), which appeared as either a fine ladder (E to G) or a ladder (H) structure. For the definition of these structures, see the text. The frequencies of these structures were counted and shown in the graphs appearing in Figures 3 to [Fig pone-0041787-g004] 5 using the legend shown in panel I of this Figure.

**Figure 3:**
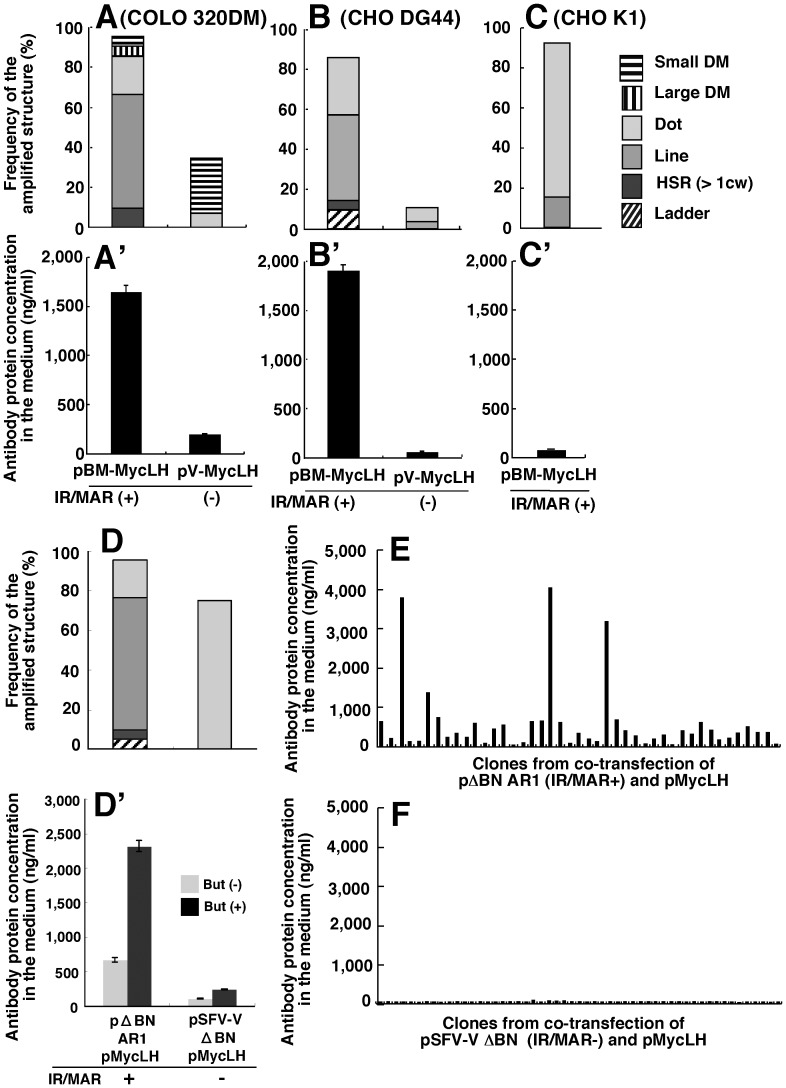
Efficient antibody production by the IR/MAR gene amplification method. COLO 320DM cells (A), CHO DG44 cells (B) and CHO-K1 cells (C) were transfected with IR/MAR-bearing pBM-MycLH or the control IR/MAR-negative pV-MycLH plasmid. CHO DG44 cells were co-transfected with IR/MAR-bearing pΔBN.AR1 and pMycLH plasmids (D, E), or co-transfected with control pSFV-V and pMycLH (D, F) plasmids. After selection of the stable transformant for about one month, gene amplification (A to C, D) and antibody expression (A’ to D’) were examined by FISH and ELISA, respectively. Clones were obtained from the cells shown in D by the limiting dilution in 96 well plates. The wells bearing a single colony were microscopically determined, and the culture liquid was analyzed by ELISA. The horizontal axis of panel E or F corresponds to 43 or 53 clones, respectively.

### Cell Culture, Transfection, Selection and Cloning

The origin and culture of human colorectal carcinoma COLO 320DM cells and hamster CHO-K1 cells have been described [7], [20]. Hamster CHO DG44 cells were obtained from Dr. Lawrence Chasin at Columbia University. These cells were grown in Ham’s F-12 supplemented with heat-inactivated 10% fetal bovine serum (FBS, EuroClone), and maintained using “TrypLE Express with Phenol Red” (Invitrogen; 12605-028).

**Figure 4 pone-0041787-g004:**
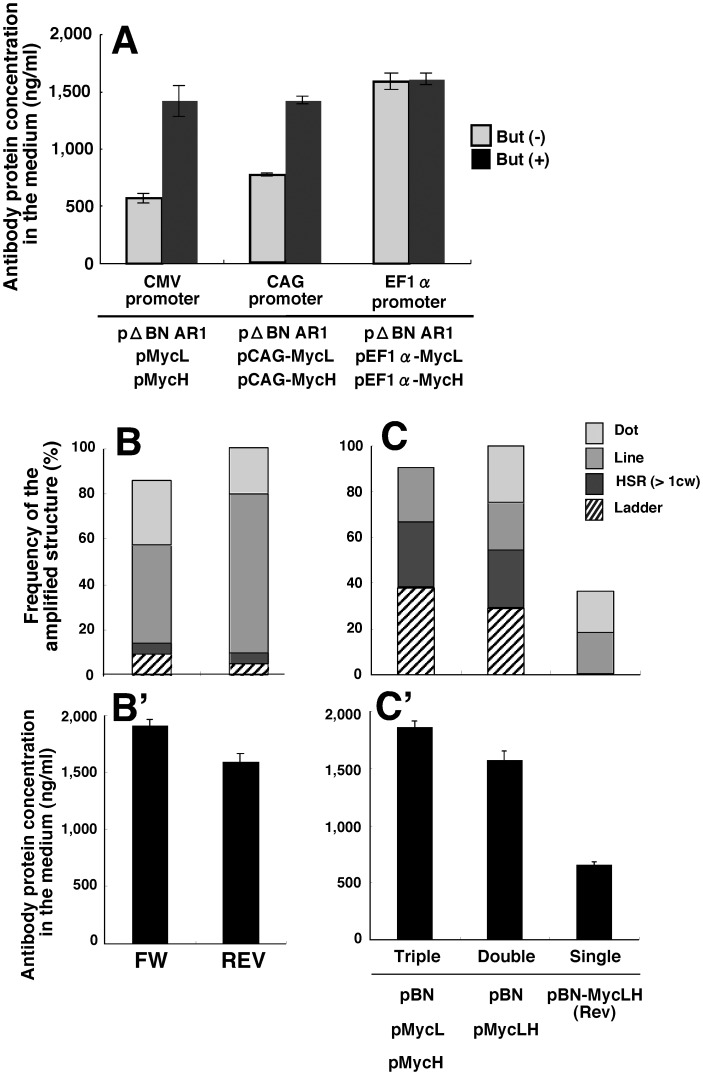
Effect of the plasmid construct and method of transfection. (A) The promoter sequences noted in the Figure were inserted on the 5′-sides of the *Ig* genes (*MycH* and *MycL*) in the constructs shown in Fig. 1A and B, and each of these plasmids was co-transfected with the IR/MAR-bearing plasmid (pΔBN AR1). (B) pBM-MycLH forward (FW) or reverse (REV), for which the orientations of the *Ig* gene were different, was also transfected. (C) Transfection with the three combinations of plasmids noted in the Figure. The plasmid was transfected to CHO DG44 cells, and the polyclonal transfectants were selected by blasticidine for about one month. The amplified structure (B, C) and the protein expression level (A, B’ and C’) were examined as in Fig. 3.

Transfection of plasmid DNA was performed using the GenePorter 2 lipofection kit (Genlantis) for the three cell lines; however, for CHO DG44 cells, most of the experiments were performed using Lipofectamine 2000 (Invitrogen; 11668-027) according to the manufacturer’s recommended protocol. Ten micrograms per milliliter blasticidin (Kaken Pharmaceuticals Co.; KK-400), 500–1000 µg/ml Geneticin 418 (Sigma), or 20 µg/ml Puromycin (Sigma) were added to the cultures beginning the day after transfection. In the case of CHO DG44 cells, the cells were detached from the plates 10 to 14 days after transfection, re-seeded in fresh plates, and cultured in the presence of 100 µg/ml blasticidin. The cells usually became confluent in 10-cm diameter dishes within 14 to 22 days after transfection and were analyzed by FISH (fluorescence in situ hybridization) and ELISA (enzyme-linked immunosorbent assay) as described below.

**Figure 5:**
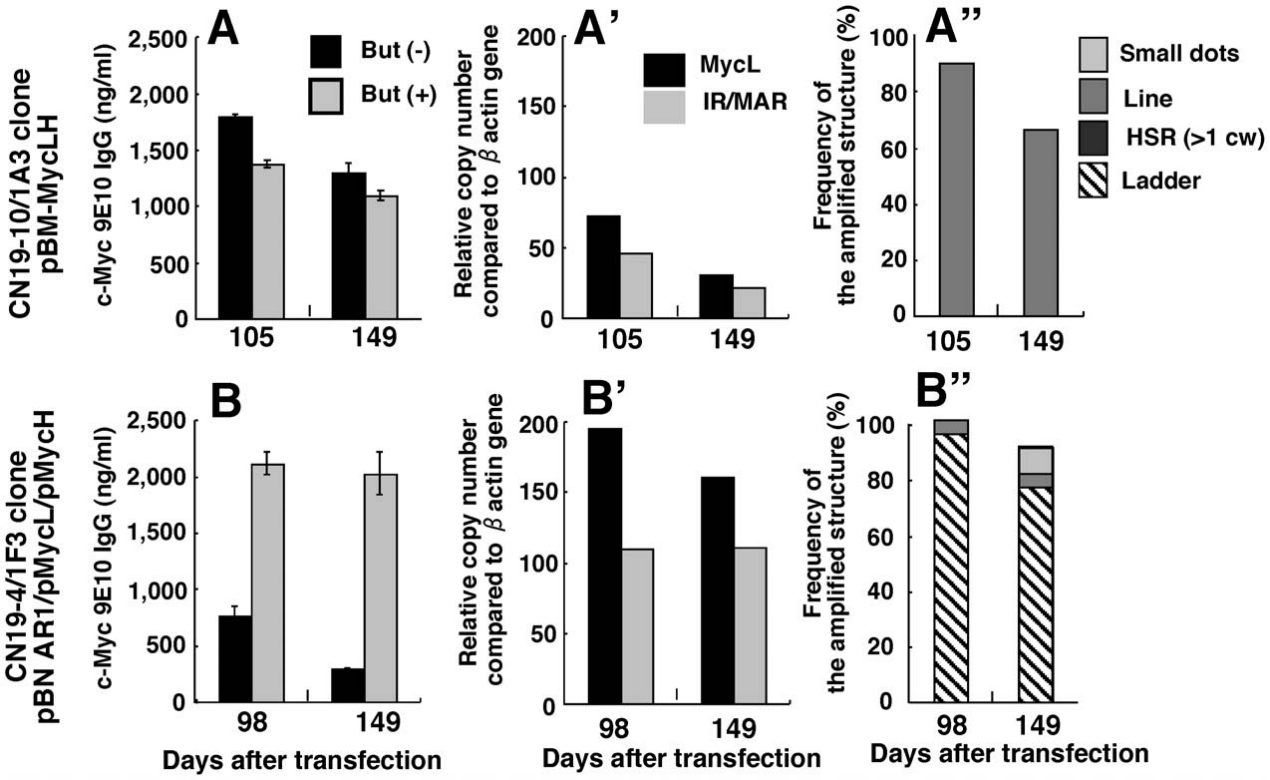
Clonal stability with respect to antibody expression and amplified genes. Two high-producer clones with amplified genes at a chromosomal line HSR (A; CN19-10/1A3) or a ladder HSR (B; CN19-4/1F3) were subjected to prolonged culture. The antibody expression in the presence or absence of butyrate (A, B), the copy number of the *Ig*-light chain (*MycL*) and the IR sequence per cell (A’, B’), and the amplified structure were examined by ELISA, real-time PCR and FISH, respectively, at the days after the plasmid transfection that were noted in the graph.

To adapt the CHO DG44-derived producer cells to serum-free suspension culture, cells growing rapidly in serum-containing medium were recovered and seeded into serum-free medium at a density of 1×10^6^ cells/ml. For serum-free medium, we used IS CHO™ (91109-1L, Irvine Scientific) containing 0.1% Pluronic F68 (GIBCO), 1 mg/mL Geneticin, and 10 µg/mL blasticidin. For adaptation, the cells were cultured in 125-ml flasks with shaking at 100 rpm, at 37°C in an atmosphere of 95% air/5% CO_2_, for three weeks. Medium was changed at three-to four-day intervals. For the production of antibody that is shown in Figure 7, the adapted cells were seeded in 20 ml of the medium, and cultured in a shaking flask. The cell density reached 1×10^6^ cells/ml after 4 days for both cell line. From this time point, we changed the entire medium every day, and the cell density reached 3×10^6^ cells/ml at day 7. After that, the entire medium was changed to the one that did not contain selection drug and contained 0.1% Pluronic F68, 10 mM HEPES with or without 5 mM butyrate, and the cells were cultured at 30°C without shaking.

**Figure 6:**
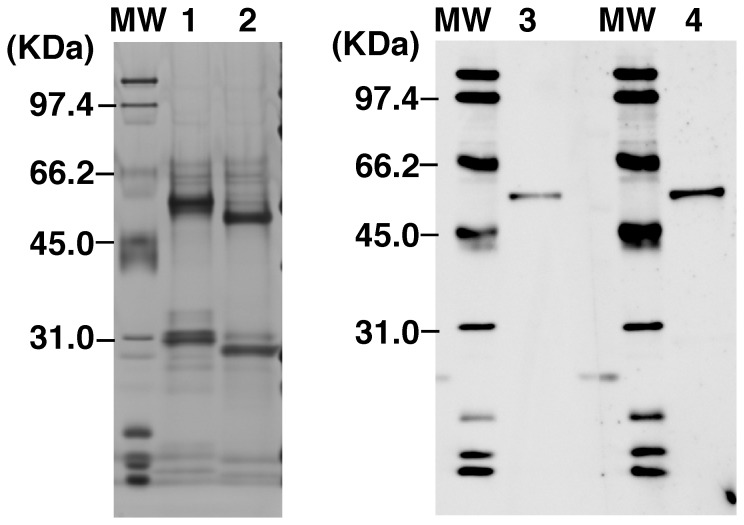
The integrity and the reactivity of the antibody produced by this method. SDS-PAGE of proteins produced by clone CN19-4/1F3. lane1, antibody purified from the clone CN19-4/1F3 culture; lane 2, commercial 9E10 c-MYC antibody; lane 3, immunoblot of the 50 kDa c-MYC fusion protein with the purified antibody from the CN19-4/1F3 culture (lane 3) or the commercial 9E10 c-MYC antibody (lane 4).

### FISH, Real-time PCR, ELISA and Biochemical Analysis of the Secreted Antibody

FISH analysis to detect the introduced plasmid sequence in metaphase chromosome spreads was performed as described previously [7], [29] using a DIG-labeled probe prepared from the introduced plasmid DNA. To measure the amount of secreted antibody, the cells were seeded in 96-well plates at a density of 1×10^5^ cells/0.2 ml in the absence of selective drug and in the presence or absence of 10 mM sodium butyrate (Sigma, B5887). After three days of culture, the supernatant was harvested and subjected to sandwich ELISA. For ELISA, we used Nunc MaxiSoap (Nunc; 442404) for the immobilization plate, rabbit anti-human IgG (The Jackson Laboratory; 309-005-082) for the immobilization antibody, purified human IgG (ZYMED; 02-7102) for the human IgG standard preparation, and horse radish peroxidase- (HRP-) conjugated anti-human IgG (The Jackson Laboratory; 309-035-082). For color development and measurement, o-phenylenediamine (OPD) tablets (Wako; 154-01673) and the iMark™ (BioRad) microplate reader were used.

**Figure 7:**
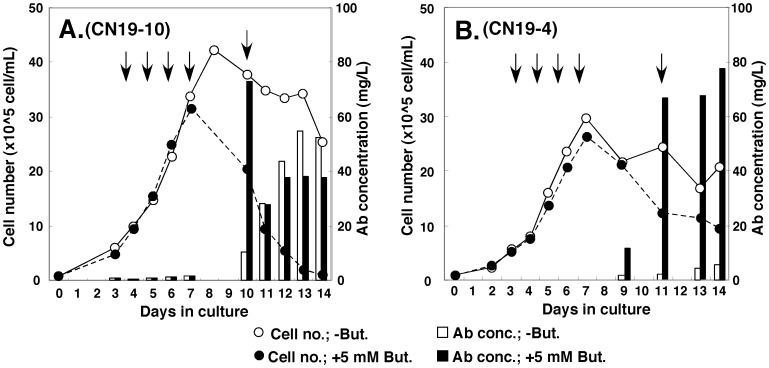
Production of antibody in suspension serum-free culture. The high producer cell clones (A, CN19-10/1A3; B, CN19-4/1F3) were adapted to suspension serum-free culture as described in the Materials and Methods section. The cell density and the antibody concentration in the medium are shown in the Graphs. The arrows indicate the time points at which the entire medium was changed.

For real-time PCR analysis, cells were harvested and genomic DNA was extracted with standard methods using SDS and proteinase K. Real-time PCR was performed on a StepOnePlus™(Applied Biosystems) system with THUNDERBIRD™ qPCR Mix (QPS-201T, Toyobo) and gene-specific primers for *β-actin*, *MycL* and IR/MAR. The relative copy numbers of *MycL* and IR/MAR were normalized to that of *β-actin*.

Recombinant antibody in the culture medium was purified using Protein A-Sepharose FF (GE Healthcare). Purified monoclonal anti-c-MYC immunoglobulin produced in mouse clone 9E10 was purchased from Sigma. These proteins were analyzed by SDS-PAGE under denaturing conditions and detected with 2D Silver Stain Reagent II (Cosmo Bio Co.). For western analysis, SDS-PAGE of a 50-kDa protein (GDF8-myc6his) containing a c-MYC protein antigenic determinant was carried out. The gel was blotted and reacted with the purified IgG from the culture supernatant and with the standard anti-c-MYC antibody 9E10 (Sigma), followed by reaction with HRP-conjugated anti-human IgG (The Jackson Laboratory, code: 309-035-082) or HRP-conjugated anti-mouse IgG (Zymed), and the band was detected using enhanced chemiluminescence (ECL) (GE Healthcare).

## Results and Discussion

### Generation of Several Types of Amplified Structures from the IR/MAR Plasmid Depending on the Cell Line Used

We previously found that the IR/MAR plasmid could be amplified and used to generate chromosomal HSR and/or extrachromosomal DMs of varying size in human colorectal carcinoma COLO 320 cells [6], [7]. However, the efficiency of amplification, the copy number per cell, and the cytogenetic manifestation of the amplified genes varied significantly depending on the cell line used, i.e. human COLO 320 [6], [7], immortalized mouse fibroblast [26], hamster CHO-K1 [20], mouse NIH3T3 [30] and human HEK293T cells [28]. To investigate IR/MAR plasmid amplification in several CHO cell sublines that are frequently used for antibody production, we transfected the cells with pBM-MycLH (Fig. 1D) coding for the antibody heavy and light chain genes with the IR/MAR sequence or pV-MycLH (Fig. 1E) coding for the antibody heavy and light chain genes without the IR/MAR sequence. We selected transfectants, prepared metaphase spreads, detected the plasmid sequence by FISH, and observed the frequency of several types of amplified structures. Representative images showing gene amplification are presented in Figure 2. As shown previously [6], the IR/MAR plasmid was amplified in COLO 320DM cells as DMs or short-to-long homogeneous HSRs (Figure 2A, 2B, and 3A). Interestingly, while the HSRs in COLO 320 cells were homogeneous arrays of the plasmid repeat without interruption of the chromosomal material (Fig. 2B and [7], [18], [25]), all of the HSRs in CHO DG44 cells appeared as a fine ladder (Fig. 2E to G), i.e., an array of tiny dots along the chromosome arm, or a ladder (Fig. 2H), where the plasmid sequences were separated by segments of unlabeled chromosomal material. In contrast, CHO-K1 cells showed little, if any, labeling of the IR/MAR plasmid (Fig. 3C); this was strikingly different from the COLO 320DM cells and also from the CHO DG44 cells. It should be noted that, although there are many CHO sublines, CHO DG44 and K1 represent two cell lines that arose directly from the ancestral CHO line [31], and therefore should have different genetic backgrounds. The genome of the K1 subline was recently sequenced [32]. Importantly, the generation of large amplified structures in both COLO 320DM and CHO DG44 cells was strongly dependent on the presence of the IR/MAR sequence on the plasmid (Fig. 3A and B). This result was reproduced in more than ten experiments using different plasmid constructs and different culture or selection conditions (data not shown).

### Dramatic Increase in Antibody Production Using the IR/MAR Plasmid

Measurement of antibody concentration in the culture medium of transfected COLO 320DM or CHO DG44 cells by ELISA showed that the IR/MAR dramatically increased antibody production (Fig. 3A’) compared to the control plasmid lacking the IR/MAR (Fig. 3B’); the protein concentration was in good agreement with the level of gene amplification (Fig. 3A and 3B). Consistent with the gene amplification level (Fig. 3C), the expression level was low in CHO-K1 cells even if the plasmid contained the IR/MAR (Fig. 3C’). We have observed a similar dramatic increase in antibody production when the IR/MAR sequence was present in several other vector constructs (data not shown).

The vector used in Figure 3A–3C contained all the antibody genes and IR/MAR in a single plasmid (Fig. 1). We have shown that any sequence can be co-amplified in the transfected cells if the DNA is co-transfected with an IR/MAR-bearing plasmid DNA [7]. Thus, we co-transfected the pMyc LH plasmid with the IR/MAR-bearing pΔBN AR1 (Fig. 1G) or the control plasmid pSFV-V ΔBN lacking the IR/MAR (Fig. 1F), and showed that gene amplification was more efficient with pMyc LH plasmid than with the plasmid pSFV-V ΔBN (Fig. 3D). Consistently, antibody production was much higher when pΔBN AR1 was employed instead of pSFV-V ΔBN (Fig. 3D’). The expression level was further increased by the addition of sodium butyrate, which inhibits histone deacetylation (Fig. 3D’). Co-transfection has the advantage that it does not require the construction of a new expression plasmid, but requires only that the plasmid coding for the gene be co-transfected with the IR/MAR-bearing plasmid. Furthermore, screening of clones obtained from the transfectants revealed that the clones showing the highest expression were obtained far more frequently with the IR/MAR-bearing plasmid (Fig. 3E) than with the control plasmid (Fig. 3F). Essentially the same results were consistently obtained using several different plasmid constructs, including those used in Figure 3A and B (data not shown).

### Effect of the Promoter that Drives the *Ig* gene

We examined whether the choice of promoter affected the protein expression level. The original CMV promoter that drives *Ig H and L* gene expression was replaced by the CAG or EF1α promoter, which has been reported to be stronger than the CMV promoter in other cells [33], [34]. Although antibody production increased slightly (Fig. 4A), there was no significant difference in antibody production between the three plasmids when sodium butyrate was added to the medium (Fig. 4A), which suggests that an epigenetic mechanism in these cells might limit gene expression.

### Effect of the Orientation of *Ig* genes

We previously reported that efficiency of gene amplification increased significantly, if the promoter-driven transcription machinery may head-on collide with the replication fork coming from the IR sequence at the MAR [7], [10]. The plasmid was less efficiently amplified if the MAR was removed, or if transcription or replication was stopped by a poly A sequence or a replication fork barrier sequence, respectively. Thus, we examined the orientation of the *Ig* gene in the pBM-MycLH plasmid. As anticipated, changes in the orientation of this construct did not result in significant changes in either the level of amplification or antibody production (Fig. 4B, 4C). This can be explained by assuming that *Ig* gene transcription was terminated by the poly A sequence and because the MAR was not located between the *Ig* genes and the IR sequence.

### Effect of the Co-transfection of Multiple Plasmid DNA

We previously reported that co-transfection, rather than transfection of a single plasmid, resulted in a higher GFP protein expression level [20], and that this could be explained by the generation of a more complex structure during co-transfection that is not readily silenced by repeat-induced gene silencing (RIG). In contrast, single transfection would generate only a simple direct repeat that is easily silenced by RIG [7], [27]. Consistent with this conjecture, the separate transfection of the IR/MAR sequence, the *Ig*-heavy chain (*MycH*), and the *Ig*-light chain (*MycL*) genes resulted in a higher amount of antibody production than that produced when all three sequences were transfected as a single construct (Fig. 4C’). This correlated with the production of ladder-type amplification in this cell line (Fig. 4C). Therefore, co-transfection also resulted in a higher expression level with this cell line and plasmid combination.

### Stability of the High-producer Clones

As described in Introduction, stability of protein expression and the amplified genes is an important issue, because the DHFR/MTx gene amplification method frequently had a stability problem. For example, antibody expression from clones obtained by this method decreased 35–92% during 36 passages [3]. We also detected similar or higher level of instability of clones obtaind from DHFR/MTx method (C. Noguchi et al., manuscript in preparation). Thus, we examined stability with respect to antibody production, *Ig* gene copy number, and the amplified structure detected by FISH. We examined two typical clones from CHO DG44 cells that bear the amplified genes as a chromosomal line HSR (CN19-10/1A3; Fig. 5A) or a ladder HSR (CN19-4/1F3; Fig. 5B), at approximately 100 days and 150 days after transfection. No significant loss of stability was observed for any of the parameters examined. This result is consistent with the results of the high-density suspension culture presented below, and indicates that this system can maintain a high protein production rate during long-term culture.

### The Quality of the Antibody Produced by the IR/MAR Gene Amplification Method

To evaluate the quality of the protein product, we purified the antibody from the culture medium of clone CN19-4/1F3 cells and analyzed it by SDS-PAGE. The result (Fig. 6 lane 1 and 2) showed that the two major two bands representing the heavy and light chains (lane 1) were slightly larger than those of the control commercial antibody (lane 2). This difference is consistent with the size of the signal peptide added to the IgG genes in our constructs. Furthermore, the purity of the antibody produced by this method was at least equal to that of the commercial antibody. Thus, we concluded that the integrity and purity of the antibody were satisfactory.

Next, we examined the reactivity of the antibodies produced. As shown in Fig. 6, the antibody recognized the 50-kDa c-MYC fusion protein (lane 4) as well as did the commercial mouse anti-c-MYC 9E10 antibody (lane 3). Thus, we conclude that the reactivity of the antibody was satisfactory.

### Specific Production Rate in Serum-free Suspension Culture

We next adapted our high-producer cell clones (CN19-10/1A3 and CN19-4/1F3) to serum-free suspension culture as described in Materials and Methods, and evaluated antibody production under these conditions (Fig. 7). Clone CN19-10/1A3 reached a higher cell density than clone CN19-4/1F3. The antibody titer was higher in the CN19-10/1A3 culture in the absence of butyrate; however, it was higher in the CN19-4/1F3 culture in the presence of butyrate. In the case of CN19-4 clone (Fig. 7B), the medium was completely changed on day 11 and, on day 13, the antibody concentration in the culture containing butyrate reached 67.7 µg/ml. The cell number was similar (12.5×10^5^ cells/ml on day 11 and 11.5×10^5^ cells/ml on day 13) and the specific production rate during this period was estimated as 29.4 pg/cell/day. Recently, much higher values (70 to 150 pg/cell/day) for the specific production rate of antibody protein were reported [3], [35], [36]. However, our value was still satisfactorily high, if we consider the quick and convenient nature of this method.

### Merit of this Method

We show that the IR/MAR gene amplification method described here provides a novel and efficient method to produce recombinant antibody. It has been designed for use in the CHO DG44 cell line, which is currently one of the most widely used lines for production of recombinant pharmaceuticals. The merits of our method over existing methods include 1) rapidity; the method requires only 2 to 3 months to obtain clonal high-producer cells. The approach is 2) convenient and involves only single-step selection and cloning, allowing simultaneous use of multiple target gene/protein combinations. The method is 3) efficient; almost all the transfected cells bear the amplified target gene, thus the frequency of high-producer cell clones is quite high. Finally IR/MAR gene amplification generates high-producer cell clones that are 4) stable over a period of months with respect to the cytogenetic appearance of the amplified region and recombinant protein production.
